# Indole-3-thio­uronium nitrate

**DOI:** 10.1107/S1600536807064707

**Published:** 2007-12-06

**Authors:** Martin Lutz, Anthony L. Spek, Erwin P. L. van der Geer, Gerard van Koten, Robertus J. M. Klein Gebbink

**Affiliations:** a Crystal and Structural Chemistry, Bijvoet Center for Biomolecular Research, Faculty of Science, Utrecht University, Padualaan 8, 3584 CH Utrecht, The Netherlands; b Chemical Biology & Organic Chemistry, Faculty of Science, Utrecht University, Padualaan 8, 3584 CH Utrecht, The Netherlands

## Abstract

In the title compound, C_9_H_10_N_3_S^+^·NO_3_^−^, the indole ring system and the thiouronium group are nearly perpendicular, with a dihedral angle of 88.62 (6)°. Hydrogen bonding generates two-dimensional networks which are linked to each other *via* π stacking inter­actions of the indole groups [average inter-planar ring–ring distance of 3.449 (2) Å].

## Related literature

For reviews of the supra­molecular chemistry of thio­urea derivatives, see: Takemoto (2005 ▶); Fitzmaurice *et al.* (2002 ▶); Schmidtchen & Berger (1997 ▶). For anion recognition of thio­uronium salts, see: Esteban Gómez *et al.* (2005 ▶). For the synthesis of the title compound, see: Harris (1969 ▶); van der Geer *et al.* (2007 ▶). For thermal motion analysis, see: Schomaker & Trueblood (1998 ▶).
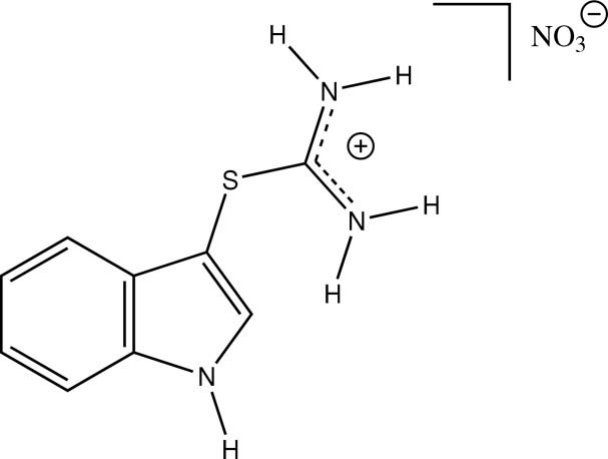


## Experimental

### 

#### Crystal data

C_9_H_10_N_3_S^+^·NO_3_^−^
*M* *_r_* = 254.27
Orthorhombic, 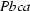

*a* = 12.0524 (2) Å
*b* = 8.7395 (1) Å
*c* = 21.1893 (3) Å
*V* = 2231.91 (5) Å^3^
*Z* = 8
Mo *K*α radiation
μ = 0.29 mm^−1^
*T* = 150 (2) K
0.30 × 0.24 × 0.06 mm

#### Data collection

Nonius KappaCCD diffractometer
Absorption correction: none
32180 measured reflections
2563 independent reflections
2120 reflections with *I* > 2σ(*I*)
*R*_int_ = 0.048

#### Refinement

*R*[*F* ^2^ > 2σ(*F* ^2^)] = 0.032
*wR*(*F* ^2^) = 0.089
*S* = 1.04
2563 reflections
194 parameters
All H-atom parameters refined
Δρ_max_ = 0.26 e Å^−3^
Δρ_min_ = −0.23 e Å^−3^

### 

Data collection: *COLLECT* (Nonius, 1999 ▶); cell refinement: *HKL-2000* (Otwinowski & Minor, 1997 ▶); data reduction: *HKL-2000*; program(s) used to solve structure: *SHELXS97* (Sheldrick, 1997 ▶); program(s) used to refine structure: *SHELXL97* (Sheldrick, 1997 ▶); molecular graphics: *PLATON* (Spek, 2003 ▶); software used to prepare material for publication: *SHELXL97*.

## Supplementary Material

Crystal structure: contains datablocks I, global. DOI: 10.1107/S1600536807064707/bt2657sup1.cif

Structure factors: contains datablocks I. DOI: 10.1107/S1600536807064707/bt2657Isup2.hkl

Additional supplementary materials:  crystallographic information; 3D view; checkCIF report

## Figures and Tables

**Table 1:** Hydrogen-bond geometry (Å, °)

*D*—H⋯*A*	*D*—H	H⋯*A*	*D*⋯*A*	*D*—H⋯*A*
N1—H1*N*⋯O1^i^	0.880 (18)	2.036 (19)	2.8290 (16)	149.4 (16)
N2—H2*N*⋯O1	0.87 (2)	2.00 (2)	2.8679 (17)	174.2 (16)
N2—H3*N*⋯O2^ii^	0.90 (2)	2.108 (19)	2.9013 (16)	147.0 (16)
N3—H4*N*⋯O2	0.90 (2)	2.00 (2)	2.8966 (19)	172.5 (19)
N3—H5*N*⋯O3^iii^	0.89 (2)	2.10 (2)	2.8817 (17)	145.0 (18)

Symmetry codes: (i) 
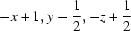
; (ii) 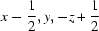; (iii) 
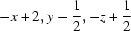
.

**Fig. 1.**
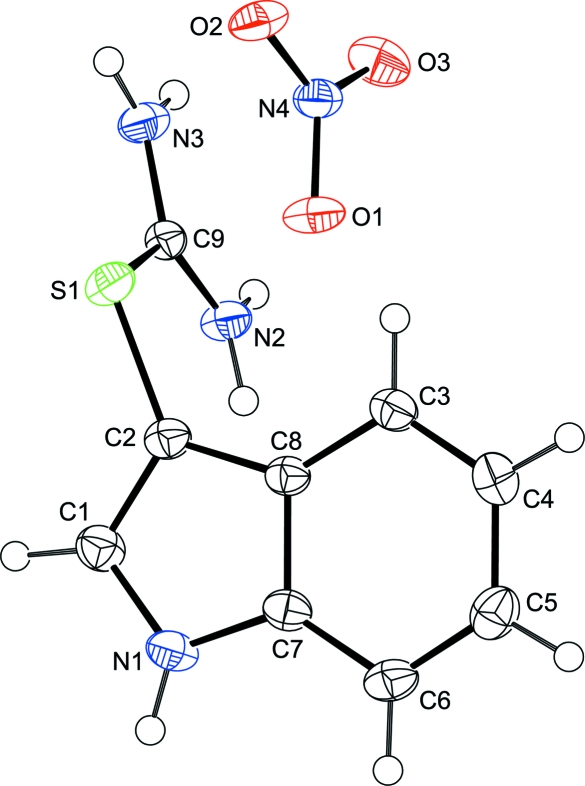
The molecular structure of (I). Displacement ellipsoids are drawn at the 50% probability level and H atoms are shown as small spheres of arbitrary radii.

**Fig. 2.**
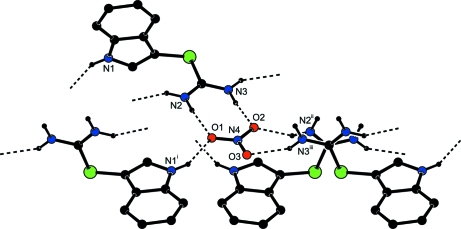
Hydrogen bonding scheme in compound (I). View along the crystallographic b axis. C—H hydrogen atoms are omitted for clarity. Symmetry operations i: 1 - x, 1/2 + y, 0.5 - z; ii: 1/2 + x, y, 0.5 - z; iii: 2 - x, 1/2 + y, 0.5 - z.

**Fig. 3.**
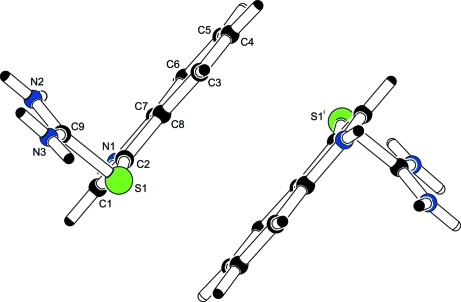
π stacking interactions between the indole ring systems in (I). View along the crystallographic a axis. Symmetry operation i: 1 - x, 1 - y, -z.

## supplementary crystallographic information

### Comment

Thiourea derivatives have found widespread application in molecular recognition and supramolecular chemistry, largely due to their hydrogen-bonding complementarity with carboxylate groups (Takemoto, 2005; Fitzmaurice *et al.*, 2002; Schmidtchen & Berger, 1997). Of all thiourea derivatives, positively-charged thiouronium salts may be among the strongest anion receptors due to their increased acidity and the electrostatic stabilization of the anion-receptor complex (Esteban Gómez *et al.*, 2005). Recently, we have demonstrated that N-substituted indole-3-thiouronium salts are readily available from indole by nucleophilic substitution at the nitrogen atom followed by electrophilic aromatic substitution with thiourea (van der Geer *et al.*, 2007). In order to gain more insight into the hydrogen-bonding properties of indole-3-thiouronium salts, we have obtained the crystal structure of the title compound indole-3-thiouronium nitrate (I).

Bond distances and angles are as expected. The thiouronium group itself is planar, with the C—N bond lengths of 1.3076 (19) and 1.3162 (19) Å indicating a significant degree of double-bond character. Reflecting the resulting hindered rotation about the C—N bonds, solution-phase ^1^H NMR shows separate signals for the thiouronium hydrogen atoms *cis* and *trans* to sulfur at room temperature. The least-squares plane of the thiouronium moiety forms an interplanar angle of 88.62 (6)° with respect to the least-squares plane of the indole group (Fig. 1).

A thermal motion analysis using the program THMA11 (Schomaker & Trueblood, 1998) results in a low weighted *R* value (*R* = SQRT[(Σ (wΔU)^2^) / (Σ (wU_obs_)^2^)]) of 0.084 indicating that the molecule behaves as a rigid body in the solid state.

All N—H groups act as hydrogen bond donors with the oxygen atoms of the nitrate anion as acceptors. O1 and O2 accept two hydrogen bonds, respectively, while O3 accepts only one. By this hydrogen bonding scheme a two-dimensional network in the a,b-plane is formed (Fig. 2).

Via π stacking interactions the indole ring systems form parallel, centrosymmetric dimers with an average ring···ring distance of 3.449 (2) Å (Fig. 3). These π stacking interactions occur between the two-dimensional hydrogen bonded layers.

### Experimental

Indole-3-thiouronium iodide was prepared as described in literature (Harris, 1969). To a solution of indole-3-thiouronium iodide (0.100 g, 0.313 mmol) in EtOH (10 ml) was added AgNO_3_ (0.0532 g, 0.313 mmol). The solution was stirred for 1 h, filtered to remove AgCl, and concentrated to approximately 3 ml. Ether (60 ml) was added, and after 48 h, white needles were collected by centrifugation, washed with ether, and dried *in vacuo*. Yield: 0.0738 g (0.290 mmol, 93%). Anal. Calcd for C_9_H_10_N_4_O_3_S: C, 42.51; H, 3.96; N, 22.03; S, 12.61. Found: C, 42.32; H, 4.08; N, 21.92; S, 12.73. The ^1^H NMR spectrum was identical to that of indole-3-thiouronium iodide. FT—IR (ATR, ν, cm^-1^): 3335, 3264, 3122, 1662, 1640, 1498, 1423, 1388, 1308, 1237, 1218, 1128, 1104, 1065, 1049, 1006, 816, 744. Crystals suitable for X-ray diffraction studies were obtained by ether vapor diffusion into an EtOH solution of the product.

### Refinement

364 frames were collected as φ scans and 397 frames as ω scans with a rotation angle of 1° and an exposure time of 60 s, respectively.

### Crystal data

**Table:**

C_9_H_10_N_3_S^+^·NO_3_^–^	*F*_000_ = 1056
*M**_r_* = 254.27	*D*_x_ = 1.513 Mg m^−^^3^
Orthorhombic, *P**b**c**a*	Mo *K*α radiation λ = 0.71073 Å
Hall symbol: -P 2ac 2ab	Cell parameters from 44477 reflections
*a* = 12.0524 (2) Å	θ = 1.0–27.5º
*b* = 8.7395 (1) Å	µ = 0.29 mm^−^^1^
*c* = 21.1893 (3) Å	*T* = 150 (2) K
*V* = 2231.91 (5) Å^3^	Plate, colourless
*Z* = 8	0.30 × 0.24 × 0.06 mm

### Data collection

**Table:**

Nonius KappaCCD diffractometer	2120 reflections with *I* > 2σ(*I*)
Radiation source: rotating anode	*R*_int_ = 0.048
Monochromator: graphite	θ_max_ = 27.5º
*T* = 150(2) K	θ_min_ = 1.9º
φ and ω scans	*h* = −15→15
Absorption correction: none	*k* = −11→11
32180 measured reflections	*l* = −27→27
2563 independent reflections	

### Refinement

**Table:**

Refinement on *F*^2^	Secondary atom site location: difference Fourier map
Least-squares matrix: full	Hydrogen site location: difference Fourier map
*R*[*F*^2^ > 2σ(*F*^2^)] = 0.032	All H-atom parameters refined
*wR*(*F*^2^) = 0.089	*w* = 1/[σ^2^(*F*_o_^2^) + (0.0479*P*)^2^ + 0.7806*P*] where *P* = (*F*_o_^2^ + 2*F*_c_^2^)/3
*S* = 1.04	(Δ/σ)_max_ < 0.001
2563 reflections	Δρ_max_ = 0.26 e Å^−^^3^
194 parameters	Δρ_min_ = −0.23 e Å^−^^3^
Primary atom site location: structure-invariant direct methods	Extinction correction: none

### Special details

**Table:**

Geometry. All e.s.d.'s (except the e.s.d. in the dihedral angle between two l.s. planes) are estimated using the full covariance matrix. The cell e.s.d.'s are taken into account individually in the estimation of e.s.d.'s in distances, angles and torsion angles; correlations between e.s.d.'s in cell parameters are only used when they are defined by crystal symmetry. An approximate (isotropic) treatment of cell e.s.d.'s is used for estimating e.s.d.'s involving l.s. planes.
Refinement. Refinement of *F*^2^ against ALL reflections. The weighted *R*-factor *wR* and goodness of fit *S* are based on *F*^2^, conventional *R*-factors *R* are based on *F*, with *F* set to zero for negative *F*^2^. The threshold expression of *F*^2^ > σ(*F*^2^) is used only for calculating *R*-factors(gt) *etc*. and is not relevant to the choice of reflections for refinement. *R*-factors based on *F*^2^ are statistically about twice as large as those based on *F*, and *R*- factors based on ALL data will be even larger.

### Fractional atomic coordinates and isotropic or equivalent isotropic displacement parameters (Å^2^)

**Table:**

	*x*	*y*	*z*	*U*_iso_*/*U*_eq_	
S1	0.69778 (3)	0.42394 (4)	0.119223 (18)	0.02623 (13)	
N1	0.37464 (10)	0.47136 (15)	0.12180 (6)	0.0261 (3)	
H1N	0.3086 (14)	0.448 (2)	0.1367 (9)	0.034 (5)*	
N2	0.67908 (11)	0.63413 (15)	0.21093 (6)	0.0247 (3)	
H2N	0.7073 (14)	0.693 (2)	0.2402 (9)	0.036 (5)*	
H3N	0.6067 (17)	0.642 (2)	0.2016 (8)	0.037 (5)*	
N3	0.85228 (11)	0.53570 (16)	0.19399 (7)	0.0281 (3)	
H4N	0.8814 (17)	0.598 (2)	0.2236 (10)	0.049 (6)*	
H5N	0.9029 (17)	0.481 (2)	0.1728 (10)	0.048 (6)*	
C1	0.47062 (12)	0.40523 (18)	0.14151 (7)	0.0263 (3)	
H1	0.4724 (13)	0.320 (2)	0.1715 (8)	0.030 (4)*	
C2	0.55884 (11)	0.47801 (17)	0.11306 (6)	0.0224 (3)	
C3	0.56104 (12)	0.70408 (16)	0.03254 (7)	0.0241 (3)	
H3	0.6404 (14)	0.7099 (18)	0.0278 (7)	0.025 (4)*	
C4	0.49126 (14)	0.80176 (18)	0.00043 (7)	0.0283 (3)	
H4	0.5191 (15)	0.876 (2)	−0.0291 (9)	0.037 (5)*	
C5	0.37534 (13)	0.79541 (18)	0.00913 (7)	0.0296 (3)	
H5	0.3302 (14)	0.868 (2)	−0.0129 (9)	0.040 (5)*	
C6	0.32737 (12)	0.68900 (18)	0.04876 (7)	0.0267 (3)	
H6	0.2499 (15)	0.6822 (18)	0.0556 (7)	0.028 (4)*	
C7	0.39845 (11)	0.58860 (16)	0.08036 (6)	0.0222 (3)	
C8	0.51463 (11)	0.59550 (15)	0.07326 (6)	0.0203 (3)	
C9	0.74591 (11)	0.54277 (16)	0.18022 (6)	0.0219 (3)	
O1	0.78687 (8)	0.82212 (13)	0.30339 (5)	0.0302 (3)	
O2	0.95458 (8)	0.75180 (13)	0.27976 (5)	0.0308 (3)	
O3	0.92651 (9)	0.94570 (13)	0.34188 (5)	0.0351 (3)	
N4	0.88984 (10)	0.84099 (14)	0.30879 (6)	0.0240 (3)	

### Atomic displacement parameters (Å^2^)

**Table:**

	*U*^11^	*U*^22^	*U*^33^	*U*^12^	*U*^13^	*U*^23^
S1	0.0208 (2)	0.0269 (2)	0.0309 (2)	0.00496 (14)	−0.00216 (14)	−0.00352 (15)
N1	0.0191 (6)	0.0331 (7)	0.0262 (6)	−0.0050 (5)	0.0008 (5)	0.0028 (5)
N2	0.0174 (6)	0.0284 (7)	0.0284 (7)	0.0015 (5)	−0.0012 (5)	−0.0036 (6)
N3	0.0179 (6)	0.0306 (7)	0.0357 (7)	0.0028 (5)	−0.0010 (5)	−0.0007 (6)
C1	0.0244 (7)	0.0292 (8)	0.0253 (7)	−0.0031 (6)	−0.0015 (6)	0.0024 (6)
C2	0.0186 (6)	0.0248 (7)	0.0239 (7)	0.0001 (5)	−0.0017 (5)	−0.0015 (6)
C3	0.0212 (7)	0.0245 (7)	0.0265 (7)	−0.0034 (6)	0.0018 (6)	−0.0024 (6)
C4	0.0323 (8)	0.0253 (8)	0.0273 (8)	−0.0035 (6)	−0.0004 (6)	0.0022 (6)
C5	0.0311 (8)	0.0286 (8)	0.0291 (8)	0.0039 (6)	−0.0066 (6)	−0.0009 (6)
C6	0.0190 (7)	0.0326 (8)	0.0287 (8)	0.0008 (6)	−0.0038 (6)	−0.0048 (6)
C7	0.0198 (7)	0.0260 (7)	0.0207 (6)	−0.0029 (6)	−0.0005 (5)	−0.0030 (5)
C8	0.0178 (6)	0.0222 (7)	0.0209 (7)	−0.0010 (5)	−0.0001 (5)	−0.0029 (5)
C9	0.0175 (6)	0.0228 (7)	0.0255 (7)	−0.0004 (5)	−0.0009 (5)	0.0059 (6)
O1	0.0145 (5)	0.0416 (6)	0.0345 (6)	0.0011 (4)	0.0000 (4)	−0.0075 (5)
O2	0.0186 (5)	0.0431 (7)	0.0308 (6)	0.0075 (5)	0.0033 (4)	0.0002 (5)
O3	0.0293 (6)	0.0403 (7)	0.0358 (6)	−0.0102 (5)	0.0002 (5)	−0.0051 (5)
N4	0.0177 (6)	0.0309 (7)	0.0235 (6)	0.0003 (5)	0.0013 (5)	0.0042 (5)

### Geometric parameters (Å, °)

**Table:**

S1—C2	1.7448 (14)	C3—C4	1.378 (2)
S1—C9	1.7566 (15)	C3—C8	1.399 (2)
N1—C1	1.359 (2)	C3—H3	0.963 (17)
N1—C7	1.3795 (19)	C4—C5	1.410 (2)
N1—H1N	0.880 (18)	C4—H4	0.963 (18)
N2—C9	1.3076 (19)	C5—C6	1.380 (2)
N2—H2N	0.87 (2)	C5—H5	0.955 (19)
N2—H3N	0.90 (2)	C6—C7	1.397 (2)
N3—C9	1.3162 (19)	C6—H6	0.946 (18)
N3—H4N	0.90 (2)	C7—C8	1.4096 (19)
N3—H5N	0.89 (2)	O1—N4	1.2572 (15)
C1—C2	1.378 (2)	O2—N4	1.2628 (16)
C1—H1	0.979 (18)	O3—N4	1.2348 (16)
C2—C8	1.432 (2)		
			
C2—S1—C9	102.23 (7)	C3—C4—H4	121.7 (11)
C1—N1—C7	109.54 (12)	C5—C4—H4	117.2 (11)
C1—N1—H1N	124.0 (12)	C6—C5—C4	121.41 (14)
C7—N1—H1N	126.1 (12)	C6—C5—H5	120.3 (11)
C9—N2—H2N	118.1 (11)	C4—C5—H5	118.3 (11)
C9—N2—H3N	122.4 (11)	C5—C6—C7	117.25 (14)
H2N—N2—H3N	119.5 (16)	C5—C6—H6	123.3 (10)
C9—N3—H4N	120.3 (13)	C7—C6—H6	119.5 (10)
C9—N3—H5N	125.3 (13)	N1—C7—C6	130.09 (13)
H4N—N3—H5N	113.9 (18)	N1—C7—C8	107.84 (12)
N1—C1—C2	109.03 (13)	C6—C7—C8	122.07 (13)
N1—C1—H1	122.8 (10)	C3—C8—C7	119.47 (13)
C2—C1—H1	128.2 (10)	C3—C8—C2	134.50 (13)
C1—C2—C8	107.55 (12)	C7—C8—C2	106.03 (12)
C1—C2—S1	125.65 (12)	N2—C9—N3	121.22 (14)
C8—C2—S1	126.55 (11)	N2—C9—S1	121.59 (11)
C4—C3—C8	118.73 (14)	N3—C9—S1	117.19 (11)
C4—C3—H3	121.4 (10)	O3—N4—O1	120.15 (12)
C8—C3—H3	119.8 (10)	O3—N4—O2	120.86 (12)
C3—C4—C5	121.04 (15)	O1—N4—O2	118.99 (12)
			
C7—N1—C1—C2	−0.19 (17)	C4—C3—C8—C7	0.1 (2)
N1—C1—C2—C8	−0.26 (17)	C4—C3—C8—C2	179.62 (15)
N1—C1—C2—S1	−174.86 (11)	N1—C7—C8—C3	178.96 (13)
C9—S1—C2—C1	−95.95 (14)	C6—C7—C8—C3	−1.2 (2)
C9—S1—C2—C8	90.45 (13)	N1—C7—C8—C2	−0.71 (15)
C8—C3—C4—C5	1.3 (2)	C6—C7—C8—C2	179.17 (13)
C3—C4—C5—C6	−1.7 (2)	C1—C2—C8—C3	−179.01 (16)
C4—C5—C6—C7	0.6 (2)	S1—C2—C8—C3	−4.5 (2)
C1—N1—C7—C6	−179.29 (15)	C1—C2—C8—C7	0.60 (16)
C1—N1—C7—C8	0.58 (16)	S1—C2—C8—C7	175.14 (11)
C5—C6—C7—N1	−179.32 (14)	C2—S1—C9—N2	4.19 (14)
C5—C6—C7—C8	0.8 (2)	C2—S1—C9—N3	−176.06 (11)

### Hydrogen-bond geometry (Å, °)

**Table:**

*D*—H···*A*	*D*—H	H···*A*	*D*···*A*	*D*—H···*A*
N1—H1N···O1^i^	0.880 (18)	2.036 (19)	2.8290 (16)	149.4 (16)
N2—H2N···O1	0.87 (2)	2.00 (2)	2.8679 (17)	174.2 (16)
N2—H3N···O2^ii^	0.90 (2)	2.108 (19)	2.9013 (16)	147.0 (16)
N3—H4N···O2	0.90 (2)	2.00 (2)	2.8966 (19)	172.5 (19)
N3—H5N···O3^iii^	0.89 (2)	2.10 (2)	2.8817 (17)	145.0 (18)

Symmetry codes: (i) −*x*+1, *y*−1/2, −*z*+1/2; (ii) *x*−1/2, *y*, −*z*+1/2; (iii) −*x*+2, *y*−1/2, −*z*+1/2.
